# The transcription factor NFIL3 controls regulatory T-cell function and stability

**DOI:** 10.1038/s12276-019-0280-9

**Published:** 2019-07-16

**Authors:** Hyeong Su Kim, Hyogon Sohn, Sung Woong Jang, Gap Ryol Lee

**Affiliations:** Department of Life Science, Sogang University, 35 Baekbeom-ro, Mapo-gu, Seoul, 04107 Korea

**Keywords:** Regulatory T cells, Lymphocyte differentiation

## Abstract

Regulatory T (Treg) cells are a CD4 T-cell subset with an important role in immune tolerance; however, the mechanisms underlying Treg cell differentiation and function are incompletely understood. Here, we show that NFIL3/E4BP4, a transcription factor, plays a key role in Treg cell differentiation and function. Microarray analysis showed that Treg cells had lower *Nfil3* expression than all other CD4 T-cell subsets. Overexpression of *Nfil3* in Treg cells led to diminished expression of *Foxp3* and other signature Treg genes, including *Il2ra*, *Icos*, *Tnfrsf18*, and *Ctla4*. Furthermore, *Nfil3*-overexpressing Treg cells exhibited impaired immunosuppressive activity in vitro and in vivo. We discovered that NFIL3 directly binds to and negatively regulates the expression of *Foxp3*. In addition, bisulfite sequencing revealed that NFIL3 induces methylation at *Foxp3* locus regulatory CpG sites, which contributes to the control of Treg cell stability. Together, these data indicate that NFIL3 impairs Treg cell function through the downregulation of *Foxp3* expression.

## Autoimmunity: Revealing the genes behind immune cell responses

The identification of a protein involved in the functioning of certain immune cells may inform future therapies for autoimmune diseases and cancer. Regulatory T cells (Treg) are immune cells that play a vital role in preventing autoimmunity, by suppressing immune responses. Abnormal Treg activity is implicated in cancer and autoimmune conditions like colitis. Gap Ryol Lee and co-workers at Sogang University, Seoul, South Korea, found that a transcription factor protein called NFIL3, coded for by the *Nfil3* gene, helps regulate Treg function and differentiation. The team found that *Nfil3* was expressed at lower levels in Treg cells than in other T cell populations. When *Nfil3* was overexpressed in Treg cells, the expression of another critical regulator gene for Treg functions was reduced, impairing the activity of the immune cells.

## Introduction

CD4 T cells play key roles in the adaptive immune system, including in the induction of cytokine production to activate various immune responses^1^. Naive CD4 T cells differentiate into Th1, Th2, and Th17 effector T cells or regulatory T (Treg) cells when they are activated via their T-cell receptors (TCRs) and a combination of cytokines. Each CD4 T-cell subset expresses a lineage-determining transcription factor that activates lineage-specific genes and drives cell differentiation. For example, T-bet is a lineage-determining transcription factor for Th1 cells (which mediate the clearance of intracellular pathogens) and induces transcription of the *Ifng* gene. The lineage-determining factor for Th2 cells, which produce interleukin (IL)-4, IL-5, and IL-13 to induce immune responses to helminths, is GATA3. Th17 cells express RORγt as a lineage-determining factor and produce the cytokines IL-17A, IL-17F, and IL-22; these cells are involved in immune responses that mediate the removal of extracellular bacteria and fungi. In addition, Th17 cells can cause autoimmune diseases, such as multiple sclerosis and rheumatoid arthritis^2^. Hence, these three subsets act as effector T cells, inducing proinflammatory reactions. In contrast, Treg cells inhibit the differentiation and proliferation of effector T cells and negatively regulate immune-mediated inflammation, controlling autoimmune diseases, and allergy; thus, Treg cells are crucial for immune homeostasis^3,4^.

Forkhead box P3 (Foxp3) is an X-chromosome-encoded Treg cell lineage-determining factor. TGF-β and IL-2 signaling induces expression of the *Foxp3* gene. IL-2 induces the JAK/STAT signaling cascade and initiates *Foxp3* transcription^5^. When TGF-β binds to TGFβR, SMAD2/3 undergoes phosphorylation and translocates to the nucleus. Phosphorylated SMAD2 binds to conserved enhancer regions, termed conserved noncoding sequences (CNSs) 1–3, at the *Foxp3* locus^6^, which contribute to the regulation of the *Foxp3* gene, along with its promoter. Each CNS contains binding sites for various transcription factors that regulate *Foxp3* expression^6–8^. CNS1 is unnecessary for thymus-derived Treg (tTreg) cell generation; however, it plays a prominent role in periphery-derived Treg (pTreg) cell formation. CNS2 has a Foxp3-binding site and contributes to Treg cell stability. Finally, CNS3, which has a c-Rel-binding site, increases Treg cell generation^9^. As a lineage-determining factor, Foxp3 activates Treg signature genes, including *Ctla4*, *Il2ra*, *Tnfrsf18*, and *Icos*. These genes participate in the suppressive function or differentiation of Treg cells. Mutation or deletion of *Foxp3* results in lymphoproliferative diseases characterized by multiorgan lymphocyte infiltration. Scurfy mice harbor *Foxp3* mutations and exhibit a severe autoimmune disorder phenotype. Similarly, immune dysregulation, polyendocrinopathy, enteropathy, X-linked (IPEX) syndrome is caused by Foxp3 dysfunction in humans^10,11^.

Nuclear factor interleukin 3 (NFIL3, also known as E4-binding protein 4, E4BP4) is a repressor of numerous genes^12^. NFIL3 contains a basic leucine zipper domain, comprising amino acids 73–146, among 462 residues; the N-terminal part of this domain directly binds to DNA, while the C-terminal region is responsible for homo- or heterodimerization of the protein. Amino acids 299–363 comprise a transcriptional repression domain^12^. NFIL3 represses genes by recruiting histone deacetylase 2 and G9a histone methyltransferase^13,14^ and regulates diverse biological processes, including the circadian rhythm, cellular viability, and hepatic metabolism^15–17^. In immune cells, NFIL3 plays a key role in B-cell IgE class switching and the development of NK cells. NFIL3 binds to the Igε promoter to stimulate IgE production^18^. *Nfil3*-deficient mice show dramatic NK cell loss due to the influence of this factor on NK cell development, maturation, and function^19^. *Nfil3*-deficient mice also exhibit elevated IL-12 p40 expression in colon tissue, which induces Th1 differentiation, resulting in spontaneous colitis^20^. Th2 cytokines are also affected by NFIL3, with increased expression of IL-5 and IL-13 in *Nfil3*^−/−^ Th2 cells^21^. Furthermore, NFIL3 links the circadian rhythm with immune cell development by suppressing the Th17-determining factor, RORγt^22^. Although its roles in the three effector T-cell subsets have been defined, the function of NFIL3 in Treg cells remains elusive.

In this study, we used an NFIL3 overexpression system to investigate the role of NFIL3 in Treg cell differentiation and function. We found that Treg cells express the lowest levels of *Nfil3* among the CD4 T-cell subsets. NFIL3 reduces *Foxp3* gene expression by binding to its promoter and CNS1–3 and by physically interacting with the Foxp3 protein. Upon overexpression, NFIL3 attenuates the suppressive ability and stability of Treg cells. Collectively, these results demonstrate that NFIL3 controls the function and stability of Treg cells.

## Materials and methods

### Mice

Six-to-eight-week-old female C57BL/6 mice were purchased from Daehan Bio Link. *Foxp3*^eGFP ^^23,24^, *Foxp3*^CRE/YFP ^^25^, and *Rosa26*^tdTomato^^ 26^ mice were purchased from the Jackson Laboratory. Mice were raised under specific pathogen-free conditions. All animal experiments were approved by the Sogang University Institutional Animal Care and Use Committee.

### Preparation and differentiation of CD4 T cells in vitro

Cell preparation and cultivation was performed as described previously^27^. Six-to-eight-week-old mice were sacrificed, and their spleens were isolated. After red blood cell lysis, cells were mixed with anti-CD25 (102014, BioLegend), anti-CD8α (100716, BioLegend), anti-I-A/I-E (107610, BioLegend), and anti-NK1.1 (108712, BioLegend) and depleted with a mixture of BioMag goat anti-mouse IgG (Qiagen) and BioMag goat anti-rat IgG (Qiagen) for negative selection. Biotinylated anti-CD62L (104404, BioLegend) and anti-biotin microbeads (Miltenyi Biotec) were used for positive selection. For activation, naive CD4 T cells were cultured with plate-bound anti-CD3ε (145-2C11) and soluble anti-CD28 (37.51). For differentiation into Th1 cells, mouse recombinant IL-2 (1 ng/ml, eBioscience), mouse recombinant IL-12 p70 (3.5 ng/ml, eBioscience), and anti-IL-4 antibody (11B11, 2 μg/ml) was added to the cell culture medium. For differentiation into Th2 cells, mouse recombinant IL-4 (5 ng/ml, eBioscience) and anti-IFN-γ antibody (XMG1.2, 2 μg/ml) was added to the cell culture medium. For Th17 cell differentiation, mouse recombinant IL-6 (50 ng/ml, eBioscience), human recombinant TGF-β1 (2 ng/ml, eBioscience), mouse recombinant IL-1β (2 ng/ml, eBioscience), mouse recombinant TNF-α (1 ng/ml, eBioscience), anti-IFN-γ antibody (2 μg/ml), and anti-IL-4 antibody (2 μg/ml) was added to the cell culture medium. For Treg cell differentiation, mouse recombinant IL-2 (1 ng/ml), human recombinant TGF-β1 (5 ng/ml), anti-IFN-γ antibody (5 ng/ml), and anti-IL-4 antibody (5 ng/ml) was added to the cell culture medium. For the in vitro plasticity assay, the media were changed to induce the indicated polarization conditions and cultured for an additional 48 h.

### RNA isolation and quantitative reverse transcription polymerase chain reaction (qRT-PCR)

Total RNA was isolated from cells using Tri-reagent (Molecular Research Center) according to the manufacturer’s instructions. RT was conducted using TOPscript RT (Enzynomics). qRT-PCR was performed using HiFast Probe Lo-ROX and HiFast SYBR Lo-ROX master mixes (PCR Biosystems Ltd.) and a Roche LightCycler 96. The sequences of primers used for quantitative PCR are listed in Supplementary Table 1.

### Flow cytometry and intracellular staining of cytokines and transcription factors

For intracellular staining, cells were harvested, fixed, permeabilized (eBioscience), and then stained with an APC-conjugated anti-Foxp3 antibody (eBioscience). For cytokine staining, cells were stimulated with PMA (Sigma), ionomycin (Sigma), and brefeldin A (BioLegend) before fixation.

### Transient reporter assay

EL4, a mouse lymphocyte cell line, was transfected by electroporation with a combination of the pCMV-*Nfil3* expression vector, the pRL *Renilla* luciferase control reporter vector, and the pGL3-*Foxp3*P reporter vector. The following day, cells were stimulated with PMA (50 ng/ml) and ionomycin (1 μM) for 4 h. Then, firefly luciferase activity was measured and normalized to that of *Renilla* luciferase.

### Retroviral transduction

Packaging cells were transfected with pMIEG3-*Nfil3* retroviral vector and pCL-eco helper vector. After 48 h, the culture supernatant, which had a high retroviral titer, was collected and filtered through a 0.4 μm filter. Naive CD4 T cells were activated for 24 h and spin infected in 1 ml of retrovirus-containing supernatant with polybrene (4 μg/ml) at 1600 × *g* for 90 min at room temperature. Cell media were changed to provide appropriate conditions, which were analyzed 48 h later.

### Coimmunoprecipitation and western blot analysis

HEK293T cells were transfected with pCMV-*Nfil3* and pCMV-*Foxp3*. After 2 days, cells were harvested, and cell lysates were isolated by sonication and then incubated with protein A/G (Santa Cruz) for 1 h to preclear nonspecific proteins. Subsequently, the cell lysates were incubated with anti-NFIL3 or anti-Foxp3 antibody overnight at 4 °C. Protein A/G was added, followed by incubation for an additional 2 h, and protein samples were washed 2–3 times with IP150 buffer. The samples were then mixed with a loading buffer and boiled for 5 min. Western blot analyses were performed as previously described^27^. Primary antibodies against NFIL3 (Cell Signaling Technology), Foxp3 (eBioscience), rabbit IgG (Cell Signaling Technology), and mouse IgG (Santa Cruz) were used.

### Chromatin immunoprecipitation (ChIP)

Treg cells (5.0 × 10^6^) were differentiated from naive CD4 T cells as described above and cross-linked with 1% formaldehyde for 10 min and then subjected to ChIP analyses using the Magna ChIP system (Merck Millipore) according to the manufacturer’s protocol. Cell extracts were incubated with anti-FLAG (Sigma) or normal mouse IgG (Santa Cruz) as a negative control. Antibody-bound protein-chromatin complexes were precipitated using magnetic protein A/G beads, washed, and eluted. Chromatin complexes were reverse cross-linked by incubation at 62 °C for 2 h. Primers used for quantifying the precipitated DNA are listed in Supplementary Table 2.

### In vitro suppression of *Nfil3*-transduced Treg cells

Naive CD4 T cells were transfected with control or *Nfil3*-hCD4 overexpression vector, followed by differentiation into iTreg cells for 48 h. hCD4^hi^ cells were sorted using a FACS Aria flow cytometer and plated in 96-well plates. CFSE (Sigma)-labeled cells (8 × 10^4^) were plated in 96-well plates with anti-CD3/CD28 beads (Invitrogen). After 3 days, CD4^+^ CD25^−^ responder T (Tresp) cells were selected and analyzed using a FACSCalibur (BD Bioscience).

### In vivo stability test

Naive CD4 T cells were isolated from *Foxp3*^eGFP^ mice and cultured under Treg-polarizing conditions. The next day, cells were transfected with control or *Nfil3*-hCD4 overexpression vector, as described above, and differentiated into iTreg cells for 48 h. hCD4^hi^ cells were sorted using a FACS Aria cytometer, and then 2.0 × 10^5^ cells were injected into 8-week-old *Rag1* KO mice. Splenocytes were isolated from *Rag1* KO mice after 1 week and analyzed using a FACSCalibur.

### RNA-sequencing (RNA-seq) and data analysis

Control and test RNA libraries were constructed using the QuantSeq 3′ mRNA-Seq Library Prep kit (Lexogen, Inc.), according to the manufacturer’s instructions. Briefly, 500 ng aliquots of total RNA was prepared, and an oligo-dT primer containing an Illumina-compatible sequence at its 5′ end was hybridized to the RNA, and RT was performed. After degradation of the RNA template, second strand synthesis was initiated using random primers containing Illumina-compatible linker sequences at the 5′ end. The double-stranded libraries were purified using magnetic beads to remove all reaction components. Libraries were then amplified, with the addition of the complete adapter sequences required for cluster generation. The completed libraries were purified from the PCR components. Single-end 75 high-throughput sequencing was conducted using the NextSeq 500 platform (Illumina, Inc., USA). QuantSeq 3′ mRNA-Seq reads were aligned using Bowtie2. Bowtie2 indices were either generated from genome assembly sequences or representative transcript sequences by alignment to the relevant reference genome and transcriptome. Alignment files were used to assemble transcripts, estimate their abundance, and detect differential gene expression. Differentially expressed genes were determined based on counts from unique and multiple alignments using coverage in Bedtools. Read count data were processed based on the quantile normalization method, using EdgeR within R using Bioconductor^28^. Gene classification was based on searches via DAVID and Medline databases (http://www.ncbi.nlm.nih.gov/).

## Results

### Treg cells have the lowest *Nfil3* expression level among CD4^+^ T-cell subsets

**Fig. 1:**
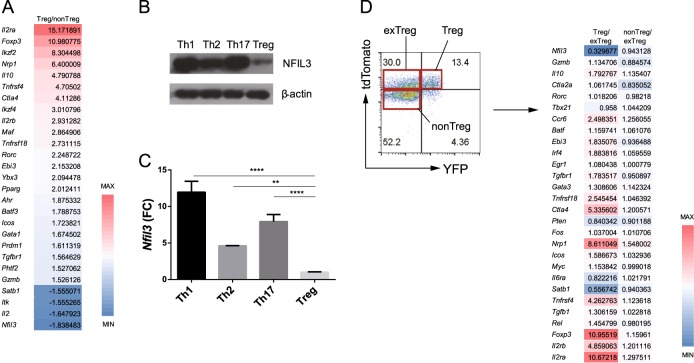
*Nfil3* expression in CD4 T-cell subsets. **a** Genes differentially expressed between Treg and non-Treg cells were analyzed by microarray analysis. Red represents increased, and blue represents decreased gene expression levels in Treg cells compared with non-Treg cells. Numbers indicate the fold-change in expression of each gene. **b** Western blot analysis of NFIL3 protein expression in each CD4 T-cell subset. Naive CD4 T cells were stimulated under Th1, Th2, Th17, and Treg-polarizing conditions for 3 days. β-Actin was used as a control. **c** *Nfil3* mRNA expression in each subset was measured by qRT-PCR. Data are representative of three independent experiments. **d** Flow cytometry analysis of splenocytes from *Foxp3*^CRE/YFP^ X *Rosa26*^tdTomato^ mice (left). Treg (#1), ex-Treg (#2), and non-Treg (#3) cells were sorted as shown. Microarray analysis data showing selected gene expression levels in each group (right). Numbers indicate the fold-change in expression of each gene. Error bars represent the standard deviation (SD). The significance of differences between groups was determined by two-way ANOVA; *P < 0.05, **P < 0.01, ***P < 0.001, and ****P < 0.0001, n.s. not significant

To search for transcription factors that affect Treg cell differentiation, we used microarray analysis to identify genes differentially expressed between Treg and non-Treg cells. For microarray analysis, CD4^+^ Foxp3^+^ and CD4^+^ Foxp3^−^ cells isolated from *Foxp3*^eGFP^ mice were sorted, and total RNA was extracted. *Nfil3* expression was significantly lower in Treg cells than in non-Treg cells (Fig. 1a). To confirm the expression of *Nfil3* in each CD4 T-cell subset, we stimulated naive CD4 T cells using Th1, Th2, Th17, and Treg-polarizing conditions and examined Nfil3 protein (Fig. 1b) and RNA (Fig. 1c) expression levels. Consistent with the microarray data, Treg cells had the lowest NFIL3 expression levels among the CD4 T-cell subsets.

To further investigate the role of Nfil3 in Treg cells, we used *Foxp3*^CRE/YFP^ X *Rosa26*^tdTomato^ tracer mice, which express Cre recombinase and a YFP reporter gene, along with *Foxp3*. *Rosa26*^tdTomato^ comprises the gene encoding the tdTomato fluorescent protein downstream of the *Rosa26* promoter, which drives constitutive expression; there is loxP-STOP-loxP between *Rosa26* and the tdTomato gene. Using these mice, current and previous *Foxp3* expression can be traced. When *Foxp3* is expressed, the Cre recombinase gene is also transcribed, and the Cre recombinase protein deletes the STOP cassette preceding the tdTomato reporter gene. Therefore, YFP^+^ tdTomato^+^ cells represent Treg cells, while YFP^−^ tdTomato^−^ cells are non-Treg cells. Treg cells can lose *Foxp3* expression and become ‘ex-Treg’ cells^29–31^. Ex-Treg cells do not express YFP; however, they do express tdTomato because the STOP cassette was deleted when *Foxp3* was previously expressed; thus, YFP^−^ tdTomato^+^ cells represent ex-Treg cells. Cells in each group were sorted, and their gene expression profiles were examined using microarray analysis. *Nfil3* was expressed at the lowest levels in Treg cells relative to non-Treg and ex-Treg cells (Fig. 1d). These results, demonstrating that *Nfil3* is differentially expressed between Treg and ex-Treg cells, prompted us to study the role of *Nfil3* in Treg cell differentiation and function.

### TGF-β signaling downregulates *Nfil3* expression

**Fig. 2:**
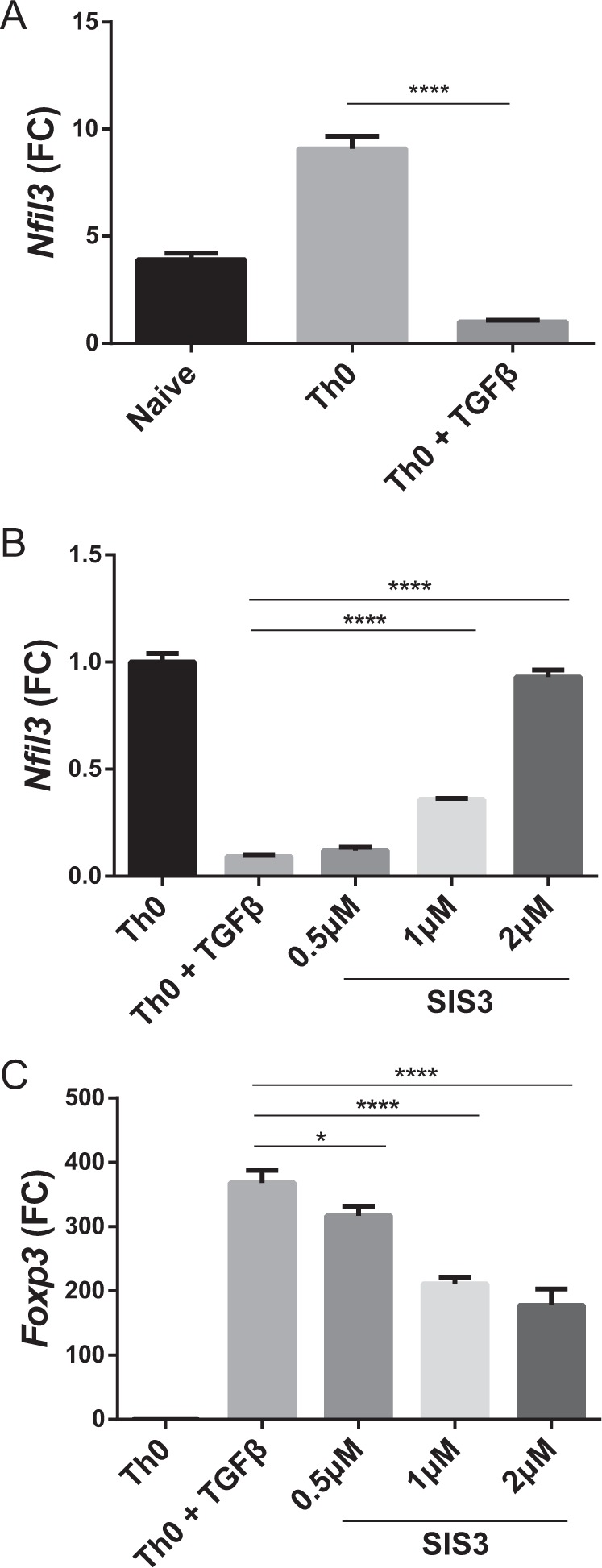
*Nfil3* expression is regulated by the TGF-β/SMAD3 signaling pathway. **a** *Nfil3* mRNA expression levels in naive CD4 T, Th0, and Th0 + TGF-β cells were determined by qRT-PCR. Cells were cultured with plate-bound anti-CD3 and soluble anti-CD28 antibody for 3 days. TGF-β was added at 5 ng/ml. Levels of *Nfil3* (**b**) and *Foxp3* (**c**) mRNA were measured by qRT-PCR. Cells were cultured with concomitant SIS3 treatment at the indicated concentrations for 3 days. qRT-PCR data are representative of three independent experiments. Error bars represent the SD. The significance of the differences between groups was determined by Student’s *t* test. *P < 0.05, **P < 0.01, ***P < 0.001, and ****P < 0.0001, n.s. not significant

As Treg cells have the lowest *Nfil3* mRNA levels among CD4 T cells, we examined the underlying signaling pathway that downregulates *Nfil3* expression in Treg cells. TGF-β is an essential cytokine for Treg cell differentiation; therefore, we hypothesized that TGF-β regulates *Nfil3* gene expression. To test this hypothesis, we compared the *Nfil3* mRNA levels among naive, Th0, and Th0 cells treated with TGF-β. Naive CD4 T cells were incubated with plate-bound anti-CD3 and soluble anti-CD28 antibodies for 2 days to stimulate their differentiation into Th0 cells. *Nfil3* expression increased in naive CD4 T cells stimulated via their TCRs, whereas it greatly decreased in response to TGF-β stimulation (Fig. 2a).

SMAD3 has previously been shown to inhibit *Nfil3* expression in NK cells^32^; therefore, we hypothesized that *Nfil3* may also be regulated by SMAD3, a component of the TGF-β signaling pathway, in T cells. To test this hypothesis, we treated cells with the SMAD3 inhibitor SIS3 and measured *Nfil3* (Fig. 2b) and *Foxp3* (Fig. 2c) expression by qRT-PCR. Inhibition of the TGF-β signaling pathway led to increased *Nfil3* expression, whereas it decreased *Foxp3* expression in a dose-dependent manner. Hence, our data suggest that *Nfil3* expression is regulated via the TGF-β signaling pathway and specifically by SMAD3.

### Overexpression of *Nfil3* downregulates *Foxp3* in Treg cells

**Fig. 3:**
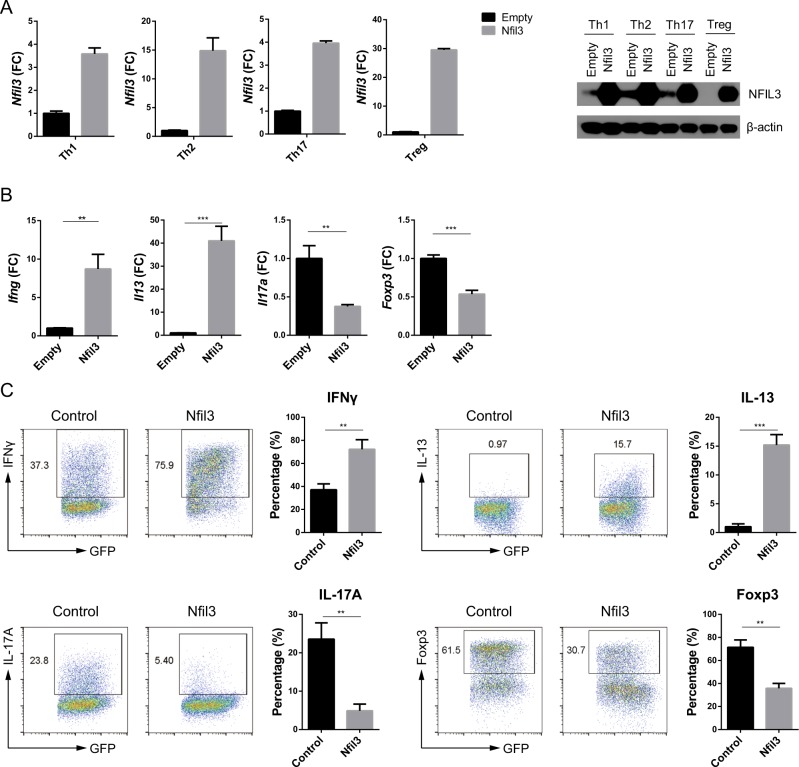
Overexpression of *Nfil3* attenuates *Foxp3* expression in Treg cells. **a** Levels of *Nfil3* mRNA in control and *Nfil3*-overexpressing CD4 T cells were determined by qRT-PCR (left panel) and western blot (right panel). Naive CD4 T cells were transduced with control (empty) or *Nfil3*-expressing vectors and polarized into each T-cell subset. Signature gene expression levels were measured in each CD4 T-cell subset by qRT-PCR (**b**), and protein levels in GFP-positive cells were evaluated by flow cytometry (**c**). Cells were cultured as described in **a**. qRT-PCR and dot plot data are representative of three individual experiments, and data in **c**, pooled from three individual experiments, were included in the statistical analysis. Error bars represent the SD. The significance of differences between groups was determined using Student’s *t* test. *P < 0.05, **P < 0.01, ***P < 0.001, and ****P < 0.0001, n.s. not significant

NFIL3 can regulate CD4 T-cell functions^20,21,33^; therefore, we investigated the role of NFIL3 in CD4 T-cell differentiation. To this end, we overexpressed *Nfil3* in each CD4 T-cell subset using the retroviral vector construct pMIEG3-*Nfil3*, which caused elevated *Nfil3* expression in all subsets tested (Fig. 3a). Upon *Nfil3* overexpression, *Ifng* and *Il13* mRNA levels increased in Th1 and Th2 cells, respectively, while those of *Il17a* and *Foxp3* decreased in Th17 and Treg cells, respectively (Fig. 3b). Consistent with the qRT-PCR data, flow cytometry analysis of protein expression showed that IFN-γ and IL-13 levels increased, while IL-17A and Foxp3 levels decreased, in response to *Nfil3* overexpression (Fig. 3c). Together, these results suggest that *Nfil3* enhances Th1 and Th2 cell differentiation, while it inhibits that of Th17 and Treg cells.

### NFIL3 represses *Foxp3* promoter activity and reduces the expression of other Treg marker genes

**Fig. 4:**
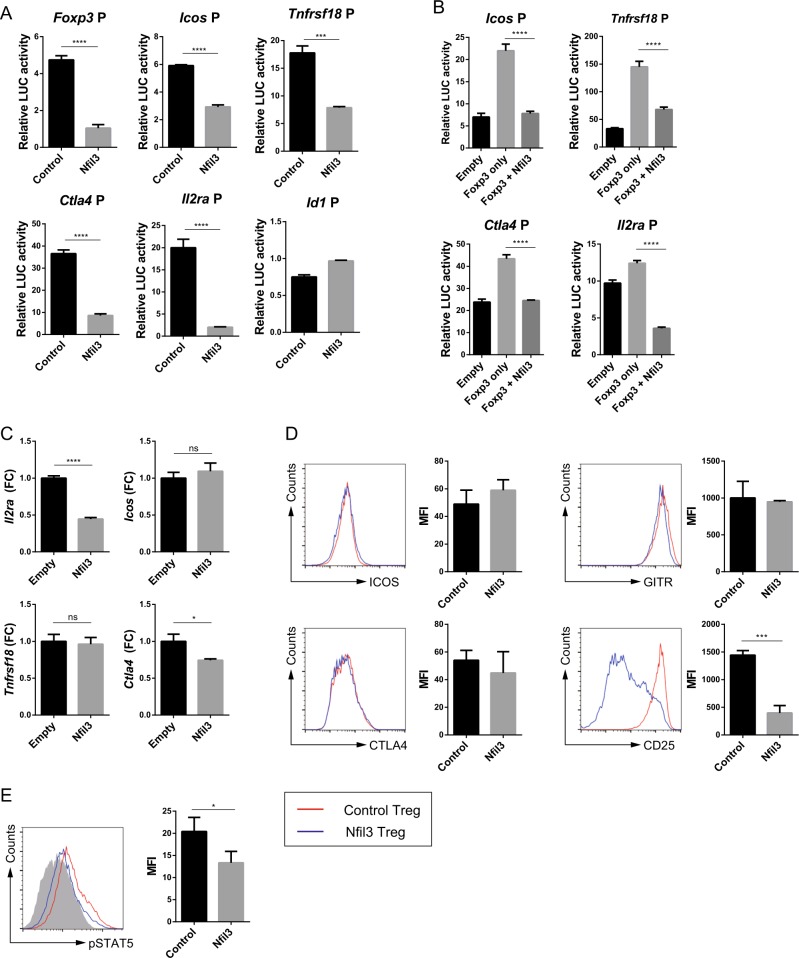
NFIL3 negatively regulates Treg signature genes. The promoter activity of each gene was measured using a transient reporter assay. **a** EL4 cells were transfected with each promoter-luciferase construct and control or *Nfil3*-expressing vectors. **b** EL4 cells were transfected as described in **a**, with the addition of cotransfection of a *Foxp3*-expressing vector. The relative luciferase activity was calculated by dividing *firefly* luciferase activity by *Renilla* luciferase activity. *Icos*, *Tnfrsf18* (GITR), *Ctla4*, and *Il2ra* (CD25) mRNA and protein expression levels were measured in control and *Nfil3*-overexpressing Treg cells by qRT-PCR (**c**) and flow cytometry (**d**). **e** pSTAT5 levels in control (empty) or *Nfil3*-overexpressing Treg cells were measured by flow cytometry. The mean fluorescence intensity (MFI) for each experiment was measured and pooled from three independent experiments (**d, e**, right panel). Transient reporter assay data are pooled from three independent experiments. qRT-PCR and dot plot data are representative of three individual experiments. Error bars represent the SD. The significance of differences between groups was determined by Student’s *t* test. *P < 0.05, **P < 0.01, ***P < 0.001, and ****P < 0.0001, n.s. not significant

To investigate how NFIL3 reduces *Foxp3* expression in Treg cells, *Foxp3* promoter activity was measured using a transient reporter assay. EL4 cells were transfected with control or *Nfil3* expression vector, along with a luciferase reporter vector containing the *Foxp3* promoter. *Foxp3* promoter activity was considerably downregulated upon *Nfil3* overexpression (Fig. 4a).

Next, we investigated the effect of NFIL3 on the expression of the Treg hallmark genes *Icos*, *Tnfrsf18*, *Ctla4*, and *Il2ra*. ICOS is an inducible costimulatory molecule involved in the expansion and maintenance of Treg cells^34^. GITR, a protein encoded by *Tnfrsf18*, plays a key role in tTreg cell differentiation and the expansion of both tTreg and pTreg cells^35^. CTLA4 promotes *Foxp3* induction in Treg cells. Finally, the *Il2ra* gene encodes the α chain of the IL-2 receptor (CD25), which is crucial for Treg cell differentiation. These genes are regulated by Foxp3 and are used as Treg markers. The promoter activity of these genes was downregulated by *Nfil3* expression (the *Id1* promoter was used as a negative control) (Fig. 4a). Therefore, these data suggest that NFIL3 selectively regulates the expression of Treg signature genes. A previous study revealed that *Foxp3* expression in EL4 cells is induced by TGF-β and TCR signaling^7^. To investigate whether NFIL3 reduces the promoter activity of these genes in a Foxp3-dependent manner, we cotransfected cells with *Foxp3* and *Nfil3* expression vectors. Consequently, the promoter activities of all four genes were elevated by *Foxp3* and attenuated by *Nfil3* expression (Fig. 4b). Hence, *Nfil3* regulates Treg signature genes via both *Foxp3*-independent and Foxp3-dependent pathways.

Next, to confirm the above results in in vitro-induced Treg (iTreg) cells, we measured the mRNA levels of the signature genes by qRT-PCR. *Nfil3* overexpression in iTreg cells reduced the transcription of *Ctla4* and *Il2ra*, but not of *Icos* or *Tnfrsf18* (Fig. 4c). Determination of the protein levels by flow cytometry showed that ICOS, GITR, and CTLA4 protein expression did not differ between control and *Nfil3-*overexpressing cells (Fig. 4d). Interestingly, the expression of CD25 was dramatically decreased in *Nfil3*-overexpressing iTreg cells (Fig. 4d). We validated this low CD25 expression by examining the phosphorylation of STAT5, which is a component of the CD25 signaling pathway. *Nfil3*-overexpressing iTreg cells had lower pSTAT5 levels than control iTreg cells (Fig. 4e). These data indicate that *Nfil3* inhibits the expression of *Foxp3* and other hallmark Treg genes, particularly *Il2ra*.

### NFIL3 binds to conserved sequences in the *Foxp3* gene and to the Foxp3 protein

The *Foxp3* gene has several CNSs that regulate its expression and Treg cell generation^9^. CNS1 participates in pTreg cell generation but not in that of tTreg cells. CNS2 regulates Treg cell stability, and both CNS1 and CNS2 are located between the *Foxp3* promoter and its first exon. CNS3, which is in intron 1, has a binding site for the c-Rel protein and enhances the expansion of the general Treg cell population.

**Fig. 5:**
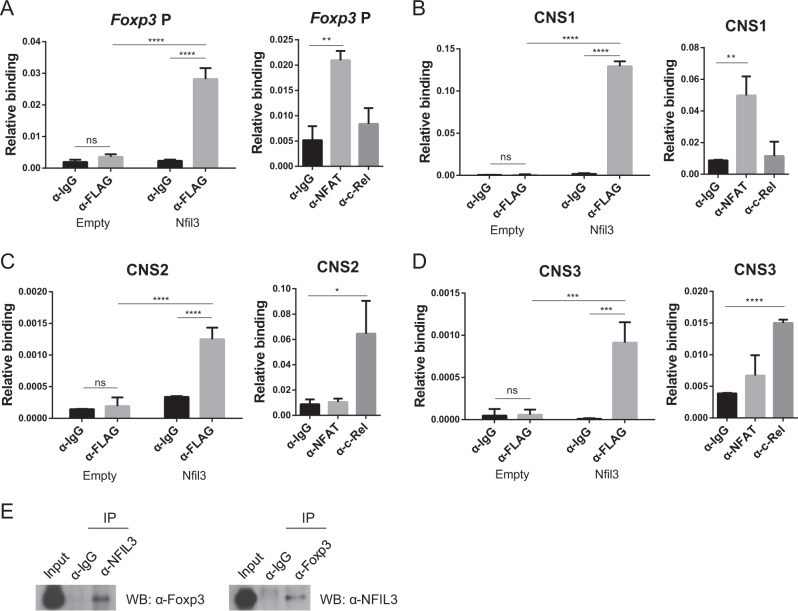
NFIL3 binds to both the *Foxp3* locus and Foxp3 protein. Binding of NFIL3-FLAG to the promoter (**a**), CNS1 (**b**), CNS2 (**c**), and CNS3 (**d**) of the *Foxp3* locus in nuclear extracts from control or *Nfil3*-Flag-overexpressing Treg cells was measured by chromatin immunoprecipitation assay. Data are pooled from three individual experiments (left panel). Binding of NFAT and c-Rel was used as the control (right panel). **e** HEK293T cells were transfected with pCMV-*Nfil3* or pCMV-*Foxp3*. Cell lysates were immunoprecipitated with anti-NFIL3, anti-Foxp3, or control IgG antibodies. Then, proteins were immunoblotted using anti-Foxp3 or anti-NFIL3 antibodies. IP, immunoprecipitation; WB western blot. Error bars represent the SD. The significance of the differences between groups was determined by two-way ANOVA. *P < 0.05, **P < 0.01, ***P < 0.001, and ****P < 0.0001, n.s. not significant

Based on our results, we hypothesized that NFIL3 may bind to the *Foxp3* gene to control its expression. To test this hypothesis, we performed a ChIP assay. Naive CD4 T cells were transfected with a control or *Nfil3*-Flag-overexpressing vector and cultured under Treg-polarizing conditions. Then, an anti-FLAG antibody was used to precipitate NFIL3. The ChIP results showed that NFIL3 binds directly to several regulatory elements in the *Foxp3* gene, including the *Foxp3* promoter, CNS1, CNS2, and CNS3 (Fig. 5a–d). Binding of NFAT and c-Rel protein to each locus was used as the control^4^.

To examine whether NFIL3 physically interacts with Foxp3, to control Foxp3 activity, we investigated protein–protein interactions between the two proteins using a coimmunoprecipitation assay and found that NFIL3 coimmunoprecipitated with Foxp3 (Fig. 5e). These data suggest that the NFIL3 protein regulates *Foxp3* expression by directly binding to *Foxp3* conserved sequences and through physical interaction with the Foxp3 protein.

### NFIL3 attenuates Treg cell stability

**Fig. 6:**
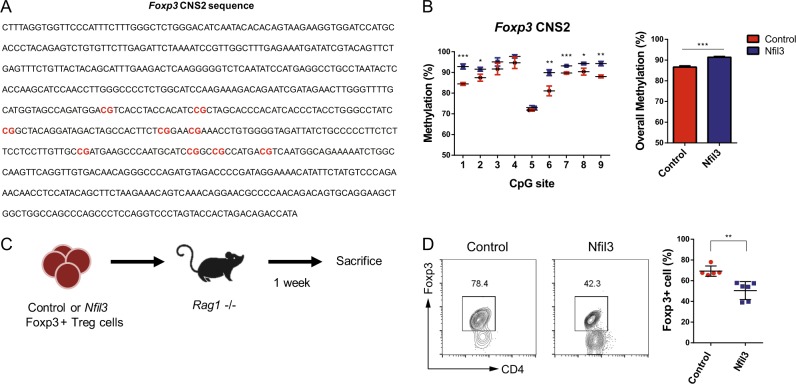
Overexpression of *Nfil3* regulates Treg cell stability. **a** Sequence of the *Foxp3* CNS2 locus. CpG sites are highlighted as red letters. **b** Methylation at each CpG site (left panel) and overall (right panel) was measured in control or *Nfil3*-overexpressing Treg cells by bisulfite sequencing. Data are pooled from three independent experiments. In vivo Treg cell stability assay. (**c**) Schematic illustration of the in vivo stability assay. Naive CD4 T cells were isolated from *Foxp3*^eGFP^ mice and transduced with empty or *Nfil3*-hCD4-overexpressing vectors under Treg-polarizing conditions. Only transfected Treg cells (eGFP^+^ hCD4^+^) were sorted. Cells (2.0 × 10^5^) were injected into *Rag1*^−/−^ mice, and after 1 week, splenocytes were isolated and analyzed. (**d**) Percentages of CD4^+^ Foxp3^+^ cells were measured in splenocytes (**c**) by flow cytometry. Representative FACS plot (left panel) and analysis of the statistical significance of differences between the two groups (right panel). Data are pooled from five to six individual experiments. Error bars represent the SD. The significance of the differences between groups was determined by Student’s *t* test. *P < 0.05, **P < 0.01, ***P < 0.001, and ****P < 0.0001, n.s. not significant

As NFIL3 reduces CD25 expression (Fig. 3), and a previous study suggested that CD25 can be used as a marker for Treg cell stability^36^, we hypothesized that NFIL3 may reduce Treg cell stability, which is regulated by two mechanisms: interleukin-2 (IL-2) signaling^36^ and CNS2 methylation^37^. As IL-2 receptor (CD25) expression was decreased by NFIL3 (Fig. 3), we next investigated methylation levels at the *Foxp3* gene CNS2 region, which contains nine CpG sites (Fig. 6a), by bisulfite sequencing. In *Nfil3*-overexpressing Treg cells, five of the nine CpG sites showed higher methylation levels than those in control Treg cells (Fig. 6b).

To further verify the loss of Treg cell stability upon *Nfil3* overexpression under physiologically relevant conditions, we performed an in vivo stability assay. Control or *Nfil3*-overexpressing Foxp3^+^ Treg cells were injected into *Rag1*^*−/−*^ mice. After 1 week, splenocytes were isolated, and the percentage of Foxp3^+^ cells among CD4 cells was determined (Fig. 6c). In the control group, Foxp3^+^ cells were maintained at a level of ~60–80%, whereas *Nfil3*-overexpressing cells were significantly reduced among the Foxp3^+^ cell population (Fig. 6d). Therefore, *Nfil3*-overexpressing Treg cells have a higher propensity to lose Foxp3 expression and their identity as Treg cells. Together, these data indicate that NFIL3 attenuates Treg cell stability.

### *Nfil3*-overexpressing Treg cells have a Th1-like gene profile

**Fig. 7:**
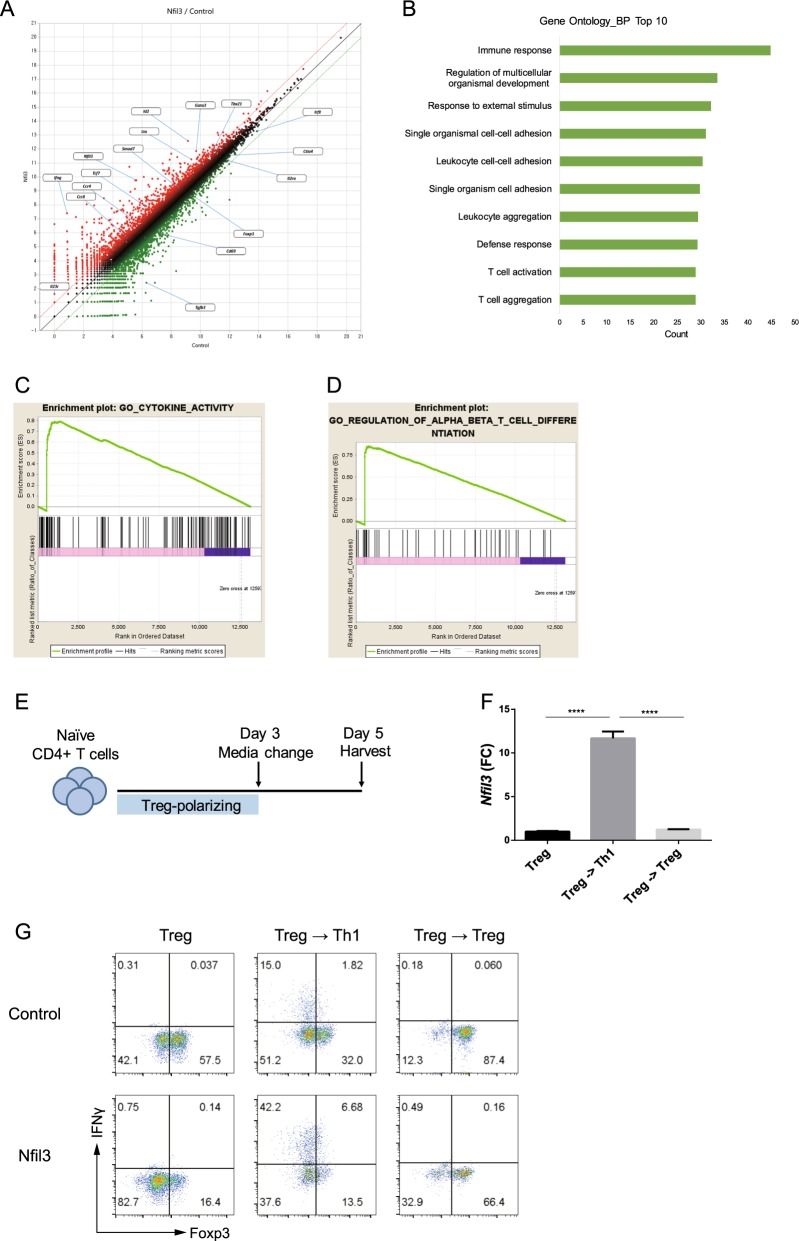
Overexpression of *Nfil3* attenuates Treg cell identity. **a** Scatterplot of RNA-seq data. RNA-seq analysis was performed using total RNA isolated from control or *Nfil3*-overexpressing Treg cells. **b** Gene Ontology analysis of genes differentially expressed in control or *Nfil3*-overexpressing Treg cells. Gene set enrichment analysis (GSEA) plots for genes in the categories cytokine activity (**c**) and regulation of α:β T-cell differentiation (**d**) in control or *Nfil3*-overexpressing Treg cells. In vitro plasticity assay. **e** Schematic of the plasticity assay. **f** qRT-PCR analysis of *Nfil3* mRNA in Treg and media-changed cells, which were cultured under Th1- or Treg-polarizing conditions, respectively. **g** Naive CD4 T cells were transduced with control or *Nfil3*-overexpressing vector on day 1, and the cells were cultured as in **e**. IFN-γ and Foxp3 levels in each cell type were measured by flow cytometry. Dot plots are representative of three individual experiments. Error bars represent the SD. Statistical differences between groups were determined by Student’s *t* test. *P < 0.05, **P < 0.01, ***P < 0.001; and ****P < 0.0001. n.s. not significant

To compare the transcriptional profiles of control and *Nfil3*-overexpressing Treg cells, we conducted RNA-sequencing (RNA-seq) (Fig. 7a). Consistent with the data above, *Nfil3*-overexpressing Treg cells had attenuated expression of Treg marker genes, including *Foxp3*, *Il2ra*, *Ctla4*, and *Tgfb3*. In addition, the expression of *Cd69*, an enhancer of Treg cell immunosuppressive function^38^, was reduced in *Nfil3*-overexpressing Treg cells. In contrast, effector T-cell-like, particularly Th1-like, gene expression was increased in *Nfil3*-overexpressing Treg cells. The expression of effector CD4 T-cell lineage-determining transcription factors *Tbx21* (encoding T-bet) and *Gata3*, the inflammatory cytokine *Ifng*, and *Lta* (TNF-β) was upregulated. A recent study revealed that ID2 contributes to the plasticity of Treg cells, manifested as their ability to become ex-Foxp3 Th17 cells^39^. Consistent with our findings that *Nfil3*-overexpressing Treg cells lose stability, gene expression profile analysis demonstrated elevated expression of the *Id2* gene in these cells. Moreover, the expression of *Smad7*, a negative regulator of TGF-β signaling^40^, was increased in *Nfil3*-overexpressing Treg cells.

We used the DAVID Gene Ontology (GO) database (http://david.abcc.ncifcrf.gov/) to analyze the biological processes that contribute to immune cell functions. The results showed highly significant enrichment of numerous genes associated with immune responses, T-cell activation, and T-cell aggregation in *Nfil3*-overexpressing Treg cells (Fig. 7b). Furthermore, gene set enrichment analysis (GSEA) showed that signature genes related to cytokine activity and regulation of T-cell differentiation were also enriched (Fig. 7c, d).

Because *Nfil3*-overexpressing Treg cells showed a Th1-like phenotype (Fig. 7a), we examined whether *Nfil3* expression is related to the plasticity of Treg cells. We cultured naive CD4 T cells under Treg-polarizing conditions for 3 days, and the cells were additionally cultured for 2 days under either Th1- or Treg-polarizing conditions (Fig. 7e). *Nfil3* expression increased when Treg cells were cultured under Th1 conditions (Fig. 7f). Interestingly, when *Nfil3*-overexpressing Treg cells were cultured under Th1 conditions, the level of IFN-γ was increased, whereas that of Foxp3 was reduced compared with the control (Fig. 7g). Collectively, our findings suggest that *Nfil3* affects the plasticity of Treg cells by causing them to acquire Th1-like gene expression and lose the expression of Treg signature genes.

### NFIL3 controls Treg cell function

**Fig. 8:**
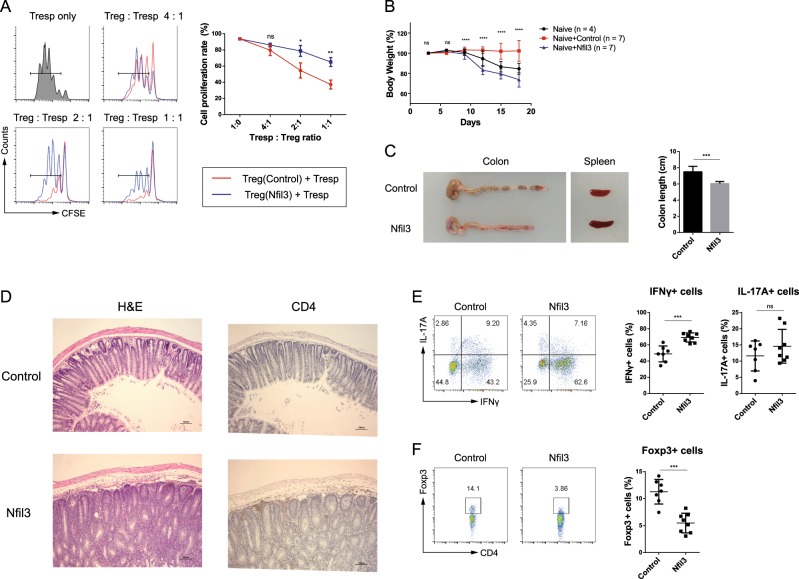
Overexpression of *Nfil3* in Treg cells impairs their immunosuppressive function. In vitro suppression activity of Treg cells was determined by measuring the proliferation of CD4^+^ CD25^−^ Tresp cells. Cells were transfected with control or *Nfil3* vector, as previously described, and GFP^+^ cells were sorted. Sorted cells were mixed with CFSE-labeled responder T (Tresp) cells at various ratios and cultured in the presence of anti-CD3/CD28 beads for additional 3 days. Tresp cells were selected and analyzed by flow cytometry. Representative flow plots from three independent experiments (left panel) and cumulative data, *n* = 3 (right panel). **b**–**f** Transfer-induced colitis model. Cells were prepared as for the in vitro suppression assay. CD4^+^CD25^−^CD62L^+^CD45RB^hi^ cells and control or *Nfil3*-overexpressing Treg cells were adoptively transferred into *Rag1* KO mice. **b** The weight of the recipient mice was measured every 3 days and is presented as the percentage of the initial weight. **c** Representative colon and spleen morphology after 18 days. Average colon length; *n* = 6–7 (right panel). **d** Hematoxylin and eosin (H&E) staining (left panel) and immunohistochemistry staining of colon sections for CD4 (right panel). Brown dots indicate CD4 T cells. Scale bars, 100 μM. Original magnification, ×100. **e** Percentage of cytokine-producing effector CD4 T cells in the mLN. **f** Percentage of Foxp3^+^ Treg cells in the mLN. Error bars represent the SD. The significance of the differences between groups was determined using Student’s *t* test. *P < 0.05, **P < 0.01, ***P < 0.001, and ****P < 0.0001, n.s. not significant

To investigate whether NFIL3 influences Treg cell function, we conducted in vitro suppression assays. Naive CD4 T cells were transfected with control or *Nfil3*-hCD4-overexpressing vector and differentiated into Treg cells. CD45.1^+^CD4^+^CD25^−^ responder T (Tresp) cells were labeled with CFSE dye. hCD4^hi^ Treg cells were mixed with CFSE-labeled Tresp cells in various ratios and incubated in the presence of anti-CD3/CD28 beads for 3 days. The proliferation of CD45.1^+^ Tresp cells was measured using flow cytometry. In the absence of Treg cells, Tresp cells actively proliferated. As the Treg cell ratio increased, Tresp cell proliferation decreased; however, when Tresp cells were cultured with *Nfil3*-overexpressing Treg cells, the inhibition of their proliferation was reduced, suggesting that *Nfil3*-overexpressing Treg cells have a diminished suppressive effect (Fig. 8a).

Next, to investigate the physiological effects of *Nfil3*-overexpressing Treg cells, we injected Treg cells intraperitoneally into *Rag1* knockout (KO) mice, along with naive CD4 T cells, to induce colitis. Compared with the transfer of naive CD4 T cells with control Treg cells (control group), the transfer of naive CD4 T cells with *Nfil3*-overexpressing Treg cells (experimental group) resulted in a dramatic decrease in mouse body weight (Fig. 8b), shortened colon length, and splenomegaly, suggesting the induction of severe inflammation (Fig. 8c). Histological analysis showed inflammation of the colonic mucosa, and immunohistochemistry revealed that the experimental group had more infiltrated CD4 T cells in the colon tissue than the control group (Fig. 8d). Next, we isolated mesenteric lymph nodes (mLNs) to analyze their IFN-γ^+^ and IL-17A^+^ cell populations (Fig. 8e). The experimental group had a higher frequency of IFN-γ^+^ cells than the control group; however, there was no difference in the frequency of IL-17A^+^ cells. Consistent with our previous findings, these results show that the *Nfil3-*overexpressing Treg cell population included a reduced frequency of Foxp3^+^ cells and had reduced *Foxp3* expression (Fig. 8f).

Overall, the transfer of naive CD4 T cells with control Treg cells successfully suppressed inflammation, whereas *Nfil3*-overexpressing Treg cells lost their suppressive activity. These results suggest that NFIL3 has a negative effect on the immune suppression ability of Treg cells, both in vitro and in vivo.

## Discussion

Treg cells repress immune responses and have beneficial effects on autoimmune and excessive immune responses; however, in some cases, for example, during chronic infectious diseases and cancer, Treg cells are detrimental to disease control. Since Foxp3 is the key transcription factor responsible for regulating the differentiation and function of Treg cells, understanding the molecular mechanisms controlling *Foxp3* expression is crucial for finding strategies to cure these immune-related diseases. Although many positive regulators of *Foxp3* have been elucidated, few negative regulators of this gene have been reported.

Here, we investigated NFIL3 as a negative regulator of Foxp3 in multiple molecular contexts. First, we determined that among CD4 T-cell subsets, *Nfil3* expression is lowest in Treg cells. Moreover, ex-Treg cells had increased *Nfil3* expression. These data indicate that NFIL3 has a deleterious effect on the stability of Treg cells; therefore, we overexpressed *Nfil3* to elucidate its effect on Foxp3. The overexpression of *Nfil3* in Treg cells led to reduced *Foxp3* expression. The molecular mechanism underlying this effect involves direct binding of NFIL3 to the CNS regions of *Foxp3* to inhibit its expression. Furthermore, NFIL3 can physically interact with the Foxp3 protein. As Foxp3 is involved in a positive feedback process that enhances its own expression^41,42^, NFIL3 may sequester the Foxp3 protein, preventing it from binding to the *Foxp3* promoter and downregulating its expression. In addition, protein–protein interactions between NFIL3 and Foxp3 could inhibit transcription of Foxp3 target genes, particularly *Il2ra*, which plays a key role in Treg cell function. The loss of *Foxp3* and Treg hallmark genes in *Nfil3*-overexpressing Treg cells reduces their immunosuppressive ability in vitro. We also observed an attenuated suppressive function of *Nfil3*-overexpressing Treg cells in a transfer-induced colitis model. Mice injected with naive CD4 T cells and *Nfil3*-overexpressing Treg cells showed signs of inflammation, including a dramatic decrease in body weight and shortened colon length. Moreover, the mice exhibited depletion of Treg cells, with a concomitant increase in IFN-γ^+^ cells. These data indicate that NFIL3 attenuates the immunosuppressive ability of Treg cells, leading to severe colitis in vivo.

Overexpression of *Nfil3* also reduced Treg cell stability. NFIL3 inhibited CD25 expression (Fig. 4c, d), and as IL-2 is essential for Treg cell differentiation, this loss of CD25 likely influences stable *Foxp3* expression. A recent study also showed that methylation of the *Foxp3* CNS2 locus is correlated with Treg cell stability^43^. Consistent with this study, we found that overexpression of *Nfil3* increased methylation at six CpG sites in the CNS2 region (Fig. 6b). One possible molecular mechanism underlying this phenomenon could be that NFIL3 recruits G9a-methyltransferase and induces a repressed chromatin state at that locus^13,14^. Loss of Treg cell stability was also observed under physiological conditions (Fig. 6d). Many studies suggest that Treg cell instability during certain inflammatory conditions can lead to their conversion to effector T-cell phenotypes^30,39,44–46^. Here, we identified that *Nfil3* expression was significantly increased when Treg cells were cultured under Th1-polarizing conditions for 2 days (Fig. 7f). This finding indicates that Treg cells enhance *Nfil3* expression intrinsically in inflammatory conditions and decrease stability to promote inflammatory conditions. In addition, *Nfil3*-overexpressing Treg cells were prone to lose Foxp3 expression and highly increase IFN-γ expression (Fig. 7g). Regarding our colitis data (Fig. 8b–f), IFN-γ-producing Th1 cells were significantly increased in the experimental group. This increase could be attributed to the loss of Treg cell stability. *Nfil3*-overexpressing Treg cells lose their suppressive function and may also convert into ex-Foxp3 Th1 cells, thereby increasing the overall population of effector T cells.

Furthermore, *Nfil3* expression is regulated by the TGF-β signaling pathway. Along with IL-2, TGF-β is an essential cytokine for Treg cell differentiation; therefore, we hypothesized that TGF-β could be a negative regulator of *Nfil3*. In addition, a previous study revealed that NFIL3-mediated NK cell development is inhibited by SMAD3, which is a signaling molecule downstream of TGF-β^32^. Our data confirm that TGF-β, along with TCR stimulation, significantly reduces *Nfil3* expression, while treatment with a SMAD3 inhibitor rescued *Nfil3* expression (Fig. 2). The reason that Th17 cells express increased levels of *Nfil3* even after treatment with TGF-β is because of the IL-6 signaling pathway. Th17 cells require IL-6 cytokines for differentiation, and a previous study discovered that IL-6/STAT3 enhances *Nfil3* promoter activity^47^. Moreover, according to our gene expression profile data (Fig. 7a), the expression of SMAD7, which negatively regulates SMAD2/3, was increased in *Nfil3*-overexpressing Treg cells^40^. From these data, we can infer that *Nfil3* may upregulate SMAD7 expression to maintain its own expression. Furthermore, as the TGF-β signaling pathway is essential for Treg cells, *Nfil3* overexpression could hinder their differentiation and maintenance. Hence, this signaling mechanism can explain how Treg cells maintain low *Nfil3* expression levels.

Since *Nfil3* is regulated by circadian rhythms, it is possible that the frequency of Treg cells could differ according to the time of the day. A recent study revealed that NFIL3 links Th17 differentiation with the circadian clock through the inhibition of RORγt^22^. As this study reveals that NFIL3 downregulates *Foxp3* expression, the development of Treg cells according to the time of the day warrants further investigation in the future.

In summary, *Nfil3* inhibits Treg cell differentiation and function by blocking *Foxp3* expression. This study reveals the molecular mechanisms underlying Treg cell differentiation and provides information that may aid in the development of therapeutic strategies aimed at curing immune-related diseases and cancer.

## Supplementary information

Supplementary Table
