# Supplementary material for: The transcription factor NFIL3 controls regulatory T-cell function and stability
Source: Exp Mol Med. 2019 Jul 16;51(7):80. doi: 10.1038/s12276-019-0280-9 (PMC6802641; doi:10.1038/s12276-019-0280-9)
Supplement: Supplementary file 1 — Supplementary Table [file 12276_2019_280_MOESM1_ESM.docx]

**Table 1. Primers used for quantitative RT-PCR**

| Primer name | Sequences (5’→3’) |
| --- | --- |
| *Ifng* Forward primer | GGATGCATTCATGAGTATTGC |
| *Ifng* Reverse primer | CCTTTTCCGCTTCCTGAGG |
| *Ifng* Probe | TTTGAGGTCAACAACCCACAGGTCCA |
| *Il13* Forward primer | GCTTATTGAGGAGCTGAGCAACA |
| *Il13* Reverse primer | GGCCAGGTCCACACTCCATA |
| *Il13* Probe | CAAGACCAGACTCCCCTGTGCAACG |
| *Il17a* Forward primer | CTCCAGAAGGCCCTCAGACTAC |
| *Il17a* Reverse primer | AGCTTTCCCTCCGCATTGACACAG |
| *Il17a* Probe | TCTGGGAAGCTCAGTGCCGCCACCAGC |
| *Foxp3* Forward primer | CCCAGGAAAGACAGCAACCTT |
| *Foxp3* Reverse primer | TTCTCACAACCAGGCCACTTG |
| *Foxp3* Probe | ATCCTACCCACTGCTGGCAAATGGAGTC |
| *Gapdh* Forward primer | CAATGTGTCCGTCGTGGATCT |
| *Gapdh* Reverse primer | GTCCTCAGTGTAGCCCAAGATG |
| *Gapdh* Probe | CGTGCCGCCTGGAGAAACCTGCC |
| *Nfil3* Forward primer | GAACTCTGCCTTAGCTGAGGT |
| *Nfil3* Reverse primer | ATTCCCGTTTTCTCCGACACG |

**Table 2. Primers used in ChIP assay**

| Primer name | Sequences (5’→3’) |
| --- | --- |
| *Foxp3* promoter  Forward primer | ATCCTCCAACGTCTCACAAACACA |
| *Foxp3* promoter  Reverse primer | TAACAGGGCTCATGAGAAACCACA |
| *Foxp3* CNS1 Forward primer | CAGAGGTCAAAAGTGTGGGTATG |
| *Foxp3* CNS1 Reverse primer | ACTTGAGTTGAGGCTAGGTTGTTC |
| *Foxp3* CNS2 Forward primer | CACATCCGCTAGCACCCACATCA |
| *Foxp3* CNS2 Reverse primer | TCATCGGCAACAAGGAGGAAGAGA |
| *Foxp3* CNS3 Forward primer | GGGGCCCACACCTCTTCTTCCTT |
| *Foxp3* CNS3 Reverse primer | CCCCCGCCACATGCCACAGTAA |
| *β-globin* Forward primer | GTTGCATTCCTCGACTGAATCCTA |
| *β-globin* Reverse primer | ACCAGTACCTGGAAGCACAATG |
| *β-globin* Probe | AAGCCACCAGCACTGTCTGCCTCAG |
